# Discovery of Loureirin analogues with colorectal cancer suppressive activity via regulating cell cycle and Fas death receptor

**DOI:** 10.1186/s40360-024-00758-2

**Published:** 2024-06-28

**Authors:** Peng Li, Xiangjuan Tian, Die Zhang, Huiping Ou, Qiufeng Huang, Wenbin Jin, Ran Liu

**Affiliations:** 1 School of Food and Drug, Shenzhen Polytechnic University, 7098 Liuxian Avenue, Shenzhen, 518055 China; 2 Key Laboratory of External Drug Delivery System and Preparation Technology in Universities of Yunnan and Faculty of Chinese Materia Medica, Yunnan University of Chinese Medicine, Kunming, Yunnan 650500 China

**Keywords:** Loureirin analogues, Chalcones and dihydrochalcones, Colorectal cancer (CRC), Cell cycle G2/M arrest, Fas

## Abstract

Chalcones and dihydrochalcones (DHCs) are important bioactive natural products (BNPs) isolated from traditional Chinese medicine. In this study, 13 chalcones were designed with the inspiration of Loureirin, a DHC extracted from *Resina Draconis*, and synthesized by classical Claisen-Schmidt reactions. Afterwards the reduction reactions were carried out to obtain the corresponding DHCs. Cytotoxicity assay indicated chalcones and DHCs possessed selective cytotoxicity against colorectal cancer (CRC) cells. The preliminary structure-activity relationships (SAR) of these compounds suggested the *α, β*-unsaturated ketone of the chalcones were crucial for the anticancer activity. Interestingly, compounds **3d** and **4c** exhibited selective anticancer activity against CRC cell line HCT116 with IC_50s_ of 8.4 and 17.9 μM but not normal cell. Moreover, **4c** could also inhibit the migration and invasion of CRC cells. Mechanism investigations showed **4c** could induce cell cycle G2/M arrest by regulating cell cycle-associated proteins and could also up-regulate Fas cell surface death receptor. The virtual docking further pointed out that compounds **3d** and **4c** could nicely bind to the Fas/FADD death domain complex (ID: 3EZQ). Furthermore, silencing of Fas significantly enhanced the proliferation of CRC cells and attenuated the cytotoxicity induced by **4c**. These results suggested **4c** exerted its anticancer activity possibly regulating cell cycle and Fas death receptor. In summary, this study investigated the anticancer activity and mechanism of Loureirin analogues in CRC, suggesting these compounds may warrant further investigation as promising anticancer drug candidates for the treatment of CRC.

**Supplementary Information:**

The online version contains supplementary material available at 10.1186/s40360-024-00758-2.

## Graphical abstract

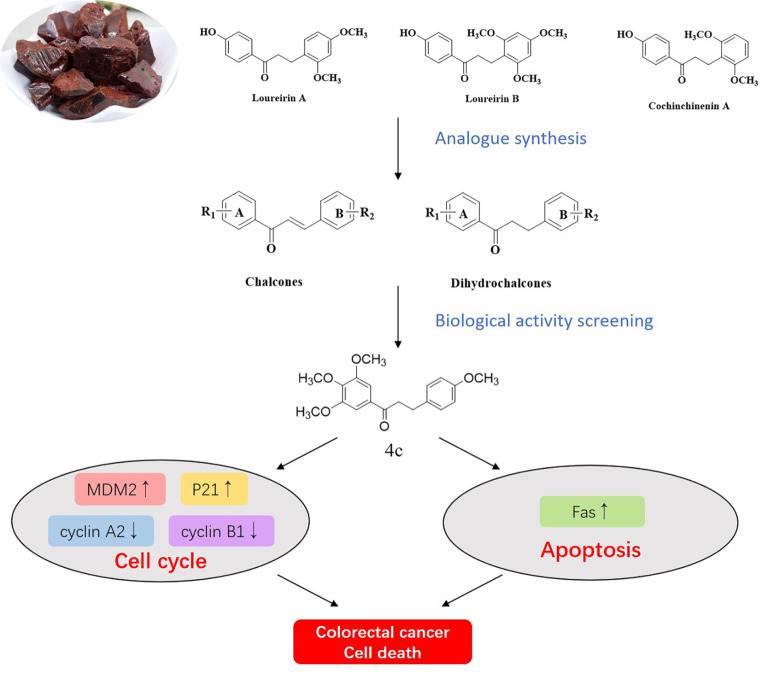

## Highlights

**•** Loureirin analogues possessed selective anti-colorectal cancer activity.
**•** Dihydrochalcone **4c** exhibited anti-cancer activity by regulating cell cycle and Fas death receptor.
**•** Silencing of FAS gene promoted colorectal cancer cell growth.

## Introduction

**Fig. 1:**
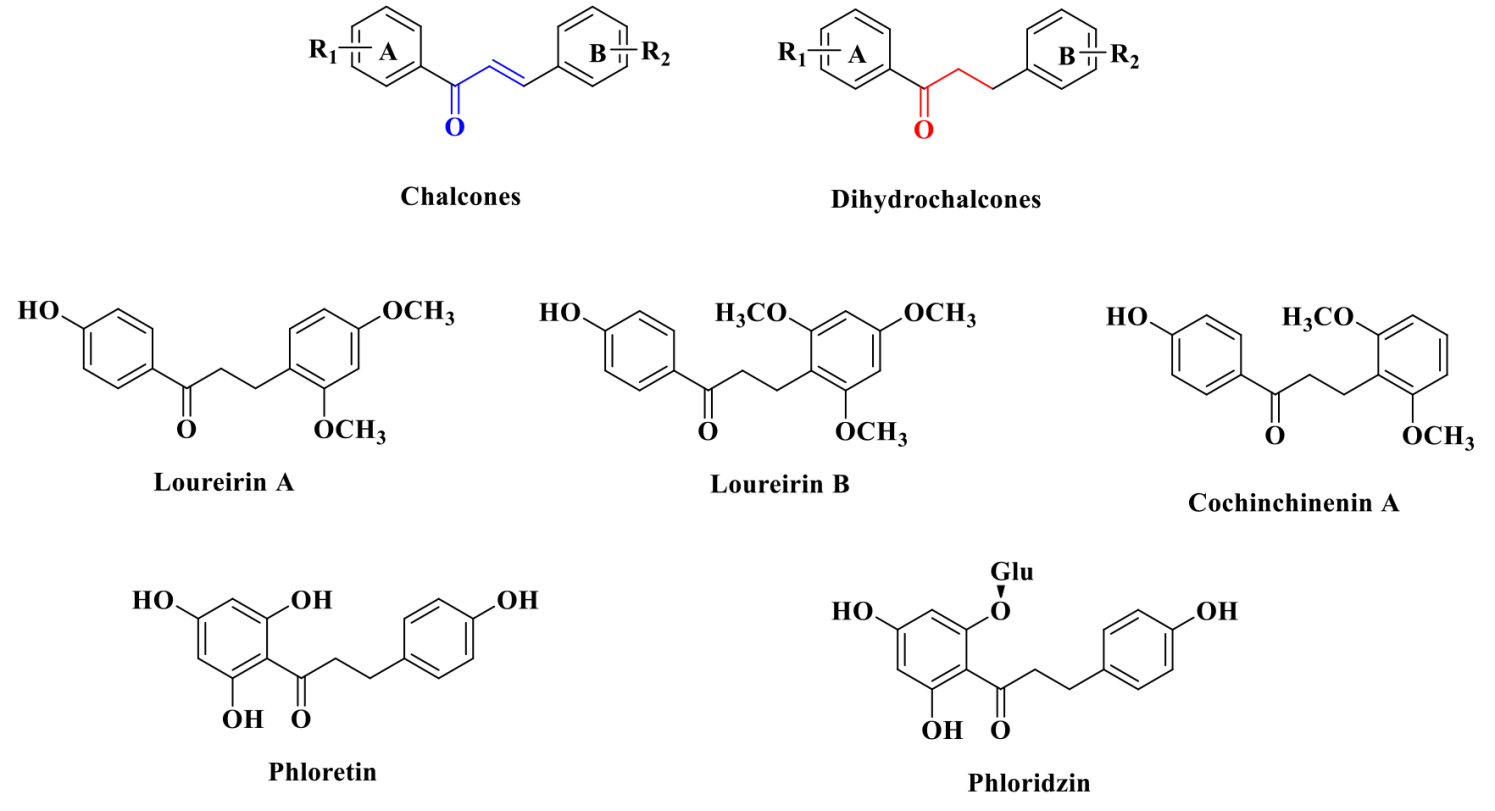
Stuctures of chalcones and Dihydrochalcones

Colorectal cancer (CRC) is the third common cancer counting for 10% of the newly diagnosed cases, leading to more than 900,000 deaths worldwide in 2020 [1]. Metastasis develops in 25–30% of CRC patients, being the leading cause of nearly 90% of the cancer-related deaths [2, 3]. Chemotherapeutic intervention coupled with surgery is the backbone of metastatic CRC treatment to increase survival [4]. Since the discovery of 5- fluorouracil (5-FU), it was the only chemotherapeutic agent available to improve 12 month survival of CRC for decades [5]. In recent years, new approached for treating CRC have been developed including combination therapy, targeted therapy and immunotherapy. In 1998 and 2002, irinotecan and oxaliplatin were approved to combine 5-FU for the treatment of advanced CRC [6]. In 2004, cetuximab was the first approved targeted therapeutic drug as an anti-EGFR agent for the treatment of CRC, followed by the emerging of numerous agents brought into preclinical and clinical trials until now [7]. In 2019, immunotherapeutic drug Nivolumab and Pembrolizumab have been approved for the treatment of metastatic CRC [8]. Mechanistically, CRC was reported to be caused by abnormal expression of genes related to DNA repair and cell cycle [9]. Anti-CRC reagents showed their anticancer activity by regulating cell cycle. Curcumin was reported to reverses 5-Fluorouracil resistance via inducing cell cycle arrest at G2/M phase [10]. Cyclin A2 and cyclin B1 were reported to be key regulators in controlling the cell entrance into M phase from G2 phase [11–13]. Metformin was also reported to alter the methylation status of tumor suppressor gene Ras association domain family 1 isoform A (RASSF1A) which induced cell cycle arrest [14]. Fas cell surface death receptor (also known as CD95), a member of the TNF receptor (TNFR) family, was reported to be involved in apoptosis pathway. Fas triggered apoptosis through binding with Fas ligands, followed by conformational changes and assembly of the Fas death-inducing signaling complex (DISC), which contains caspase-8 and activates the caspase cascade [15, 16]. Unfortunately, the putative clinical use of Fas ligand as anticancer reagent was troubled by severe liver toxicity related to the high expression of Fas in hepatocytes. Ligand-independent activation of Fas suggested that the death receptor could also be activated intracellularly in the absence of Fas ligand. This provided a novel framework for the development of anticancer drug targeting Fas [17]. Chalcones bearing two aromatic rings linked by an α, β-unsaturated propenone linker were considered to be primary precursors in the synthesis of flavonoids which were privileged scaffolds widely used for drug discovery in medicinal chemistry [18, 19]. The reactive α, β-unsaturated ketone was not only associated with the pharmacological properties including antimicrobial, antioxidant, anti-inflammatory and antitumor activities etc., but was also responsible for the key steps in which chalcones could be reductive to dihydrochalcones (DHCs) [20–23]. DHCs chemically distinct from chalcones also had two aryl rings connected through a saturated three-carbon bridge [24]. To date, DHCs for instance Phloretin, Phloridzin and Loureirin etc. shown in Fig. 1 have received growing attentions not only due to their convenient synthesis but also because of their broad functional and health-endorsing properties [25–27]. Loureirin A, Loureirin B, and Cochinchinenin A belonging to DHCs were important bioactive components extracted from traditional Chinese medicine *Resina Draconis* [28]. Several research results suggested that Loureirin analogues showed promising antiproliferative activities against a collection of human cancer cell lines. In this study, we conducted the anticancer activity screening and mechanism investigation of a series of chalcones and Loureirin analogues made by ourselves in CRC.

## Results

### Chemistry

**Scheme 1:**
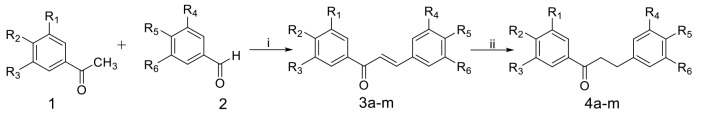
Reagents and conditions: (i) EtOH, 20%NaOH, 0℃, 8 h; (ii) EtOH, Acetone, H_2_, 10%Pd/C, HCO_2_NH_2_

Claisen-Schmidt aldol condensation of acetophenone with the corresponding aromatic aldehyde in the presence of aqueous NaOH in ethanol gave the chalcone products. Dihydrochalcones were obtained by regioselective reduction of carbon–carbon double bond in α, β -unsaturated ketones. One of the methods used gaseous hydrogen, of which addition to the α, β-unsaturated olefinic bond was catalyzed by ruthenium salts in dioxane. Other methods used such common catalysts such as palladium, nickel and iridium. The chalcones **3** were prepared in good yields using the Claisen–Schmidt condensation of ketones **1** and aldehydes **2** (Scheme 1). This method for the preparation of chalcones was attractive since it predominantly generated the trans conformer from simple building blocks. A large number of these substituted benzaldehydes and acetophenones were commercially available and inexpensive. A series of DHCs **4** were prepared by base promoted reaction of chalcones **3** with palladium-carbon system in a hydrogen atmosphere. These products were easy to separate by flash chromatography (Scheme 1).

**Table:** 

Compound	R_1_	R_2_	R_3_	R_4_	R_5_	R_6_
**4a**	H	OCH_3_	H	H	OCH_3_	H
**4b**	OCH_3_	OCH_3_	H	H	OCH_3_	H
**4c**	OCH_3_	OCH_3_	OCH_3_	H	OCH_3_	H
**4d**	F	H	F	H	OCH_3_	H
**4e**	CH_3_	F	H	H	OCH_3_	H
**4f**	H	OCH_3_	H	OCH_3_	OCH_3_	H
**4g**	OCH_3_	OCH_3_	H	OCH_3_	OCH_3_	H
**4h**	OCH_3_	OCH_3_	OCH_3_	OCH_3_	OCH_3_	H
**4i**	CH_3_	F	H	OCH_3_	OCH_3_	H
**4j**	H	OCH_3_	H	OCH_3_	OCH_3_	OCH_3_
**4k**	OCH_3_	OCH_3_	H	OCH_3_	OCH_3_	OCH_3_
**4l**	OCH_3_	OCH_3_	OCH_3_	OCH_3_	OCH_3_	OCH_3_
**4m**	CH_3_	F	H	OCH_3_	OCH_3_	OCH_3_

### Anticancer activity screening of chalcones 3a-m and DHCs 4a-m

**Fig. 2:**
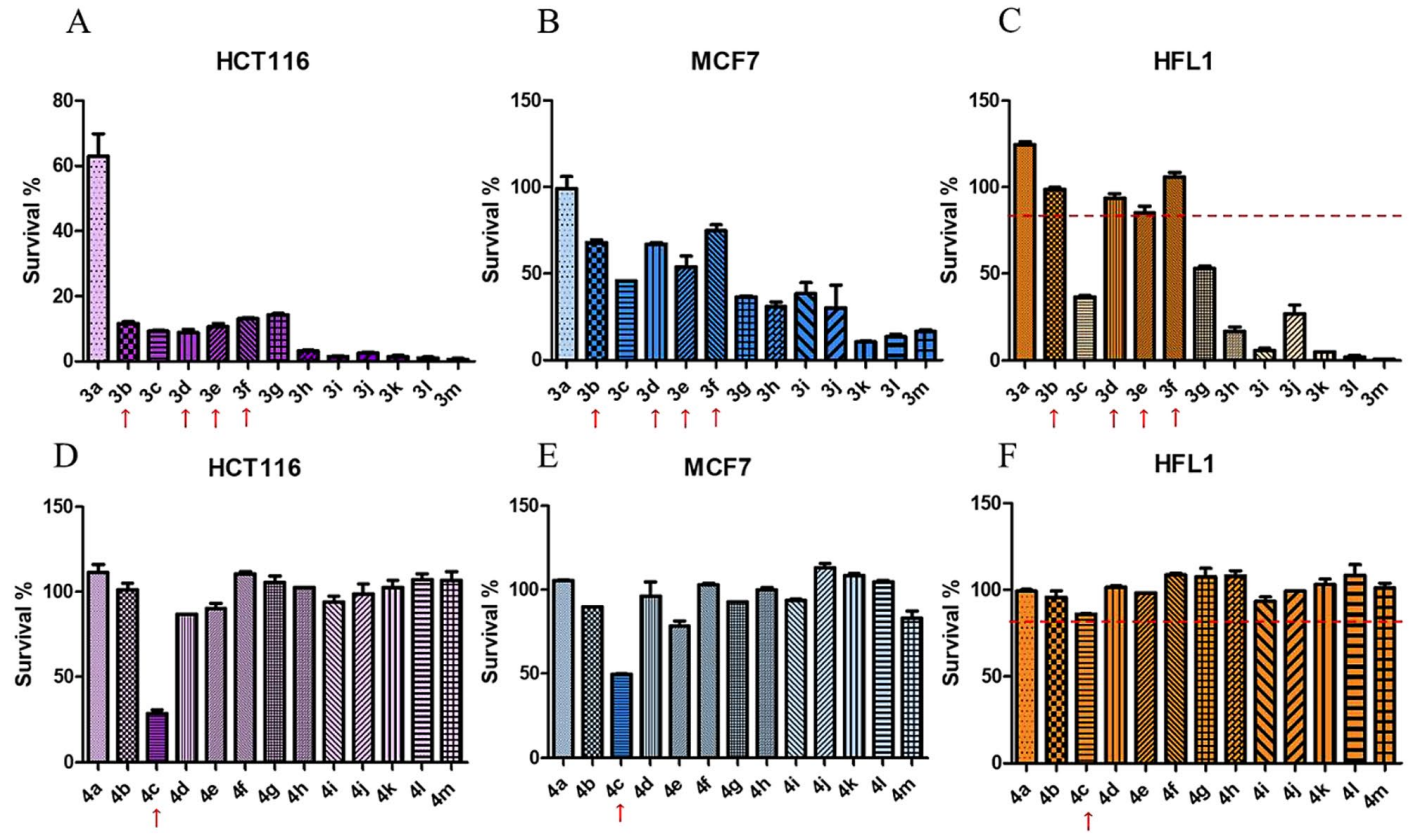
Anticancer activity screening of chalcones 3a-m and DHCs 4a-m. **A**–**F**, CCK-8 assay of cancer cells and normal cell with 20 μM of chalcone treatment

The anticancer activity of chalcones **3a-m** and DHCs **4a-m** was preliminary screened by CCK-8 assay at a concentration of 20 μM. Figure 2 showed the cytotoxicity of chalcones **3a-m (A–C)** and DHCs **4a-m (D–F)** against CRC cell line HCT116, breast cancer cell line MCF7 and human fetal lung fibroblast1 HFL1, respectively. The results indicated that all chalcones **3** (Fig. 2A) with α, β-unsaturated ketone showed higher anticancer activity in HCT116 cell line compared to DHCs **4** (Fig. 2D) with saturated ketone. Similar results could also be found in MCF7 cell line as shown in Fig. 2B and E. Moreover, chalcones **3** exhibited much higher anticancer activity against CRC cell line HCT116 than breast cancer cell line MCF7 (Fig. 2A, B). Herein, we used human fetal lung fibroblast HFL1 [29–31] to evaluate the cytotoxicity of chalcones **3** against normal cell (Fig. 2C). Surprisingly, chalcones **3a, 3b, 3d, 3e** and **3f** were shown to be non-cytotoxic against HFL1 with a percentage survival rate higher than 80%. However, chalcones **3a** displayed non-cytotoxicity toward both HCT116 and MCF7 cells, either. Encouragingly, DHC **4c** was suggested to be cytotoxic to both HCT116 and MCF7, but not to HFL1, unlike other DHCs **4** without anticancer activity (Fig. 2D–F). Taken together, chalcones **3b, 3d, 3e, 3f** and DHC **4c** were selected for further biological activity assay.

**Fig. 3:**
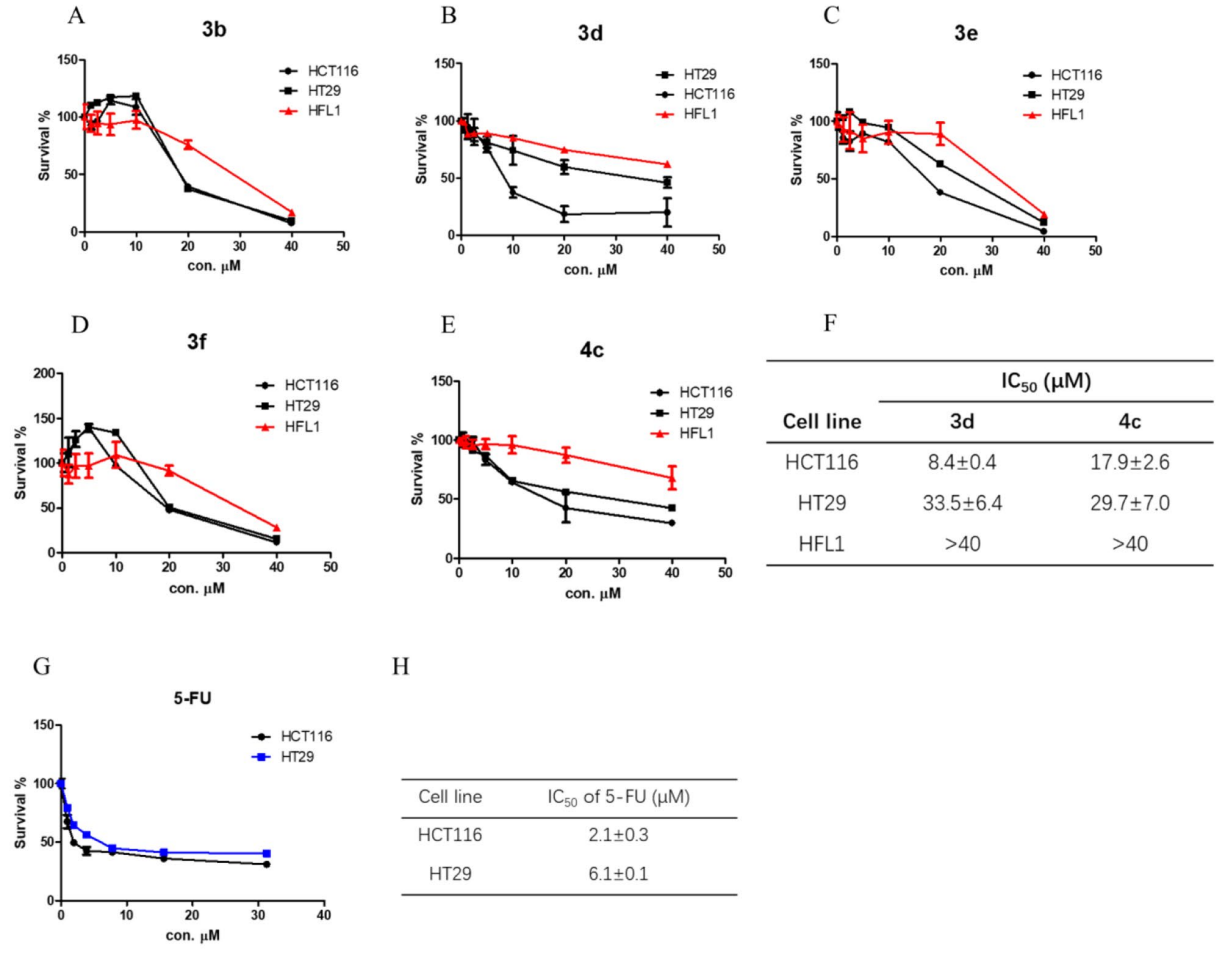
Anti-proliferative activity screening of chalcones 3 and DHCs 4. **A**–**E**, CCK-8 assay of CRC cells and normal cell with series dilution of chalcones. **F**, IC_50_ of **3d** and **4c** against CRC cells and normal cell. **G**–**H**, CCK-8 assay and IC_50_ of the positive control drug 5-FU. Data were presented as mean ± SD

According to the preliminary screening assay, the anticancer activity of chalcones were much more potent against CRC cells compared to breast cancer cells. Therefore, we further determined the cytotoxicity of compounds **3b, 3d, 3e, 3f** and **4c** against two CRC cell lines HCT116 and HT29, and human fetal lung fibroblast HFL1 as well. Among these compounds, the results suggested that the anti-proliferative activity of compounds **3d** and **4c** showed much more specific to CRC cells compared to normal cell HFL1 at 10, 20 and 40 μM (Fig. 3). As shown in Fig. 3F, the anti-proliferative activity of compounds **3d** and **4c** against HCT116 with IC_50_ of 8.4 μM and 17.9 μM respectively was much more potent compared with that against HT29 and normal cell line HFL1. It also indicated that **3d** exhibited more promising anti-CRC effects than the positive control cisplatin with IC_50_ value of 14.4 μM according to the dataset provided by Genomics of Drug Sensitivity in Cancer website (https://www.cancerrxgene.org/). However, **3d** the antitumor activity of **3d** was not as potent as that of 5-Fu as shown in Fig. 3G, H.

### Compounds 3d and 4c inhibited the growth and migration of CRC cells

**Fig. 4:**
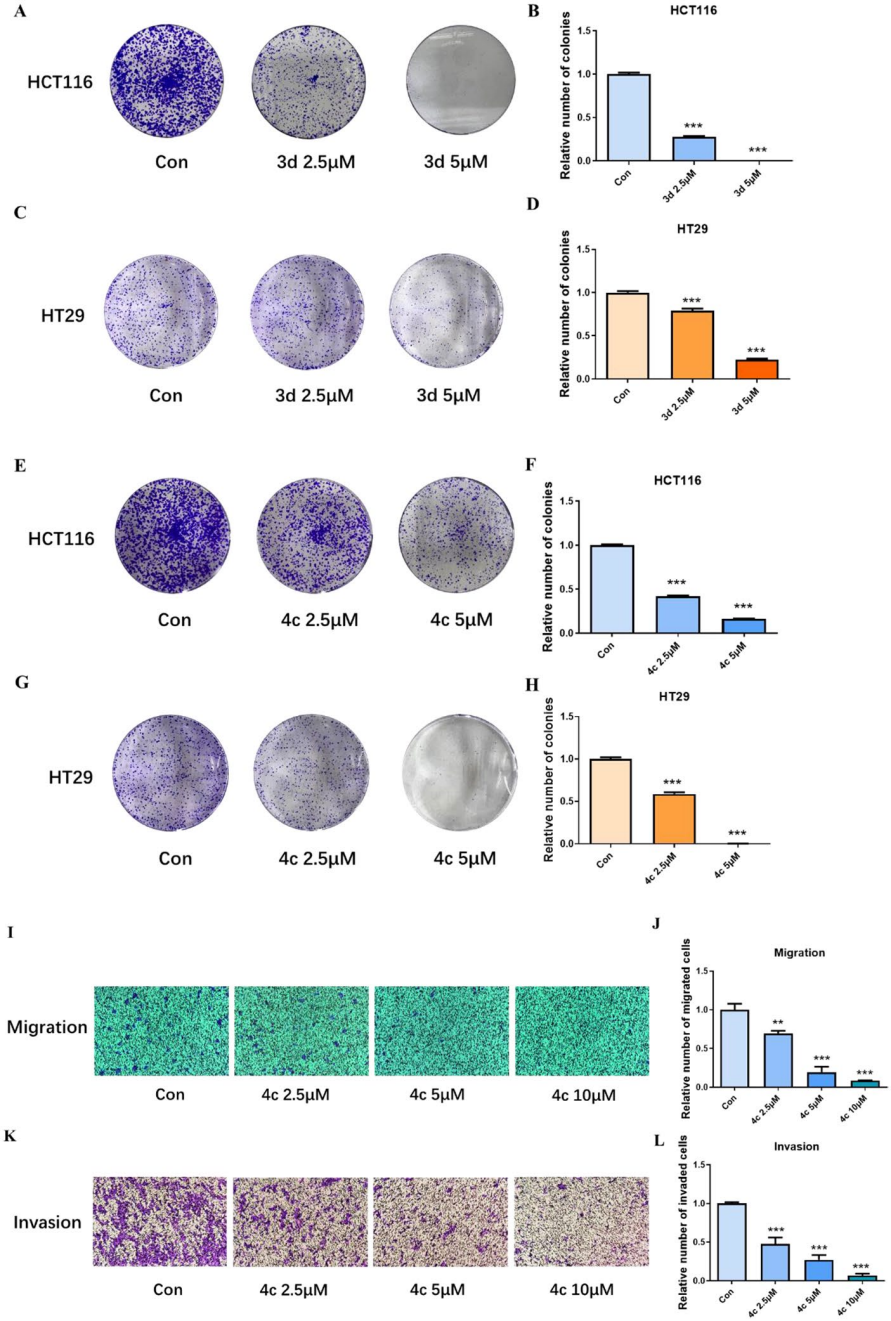
Compounds 3d and 4c inhibited the growth and migration of CRC cells. **A**–**D**, colony formation assay of HCT116 and HT29 treated with **3d**. **E**–**H**, colony formation assay of HCT116 and HT29 treated with **4c**. **I, J**, transwell migration assay of HCT116 cell. **K, L**, transwell invasion assay of HCT116 cell. Data were presented as mean ± SD. Statistically significant differences were indicated as *P < 0.05, **P < 0.01, ***P < 0.001

Next, we determined the effects of compounds **3d** and **4c** on colony formation, cell migration and invasion. As shown in Fig. 4A–D and Fig. 4E–H, both **3d** and **4c** inhibited the colony formation of HCT116 and HT29 cells, respectively. Furthermore, Fig. 4I–L indicated that **4c** could inhibit the transwell migration and invasion of HCT116 cell. Compound **3d**, however, showed no effect on either the migration or the invasion of HCT116 cell at the same condition (data not shown). These results indicated that compound **4c** could inhibit both the cell growth and migration of CRC cell HCT116.

### RNA-seq indicated compound 4c regulated p53 signaling pathway

**Fig. 5:**
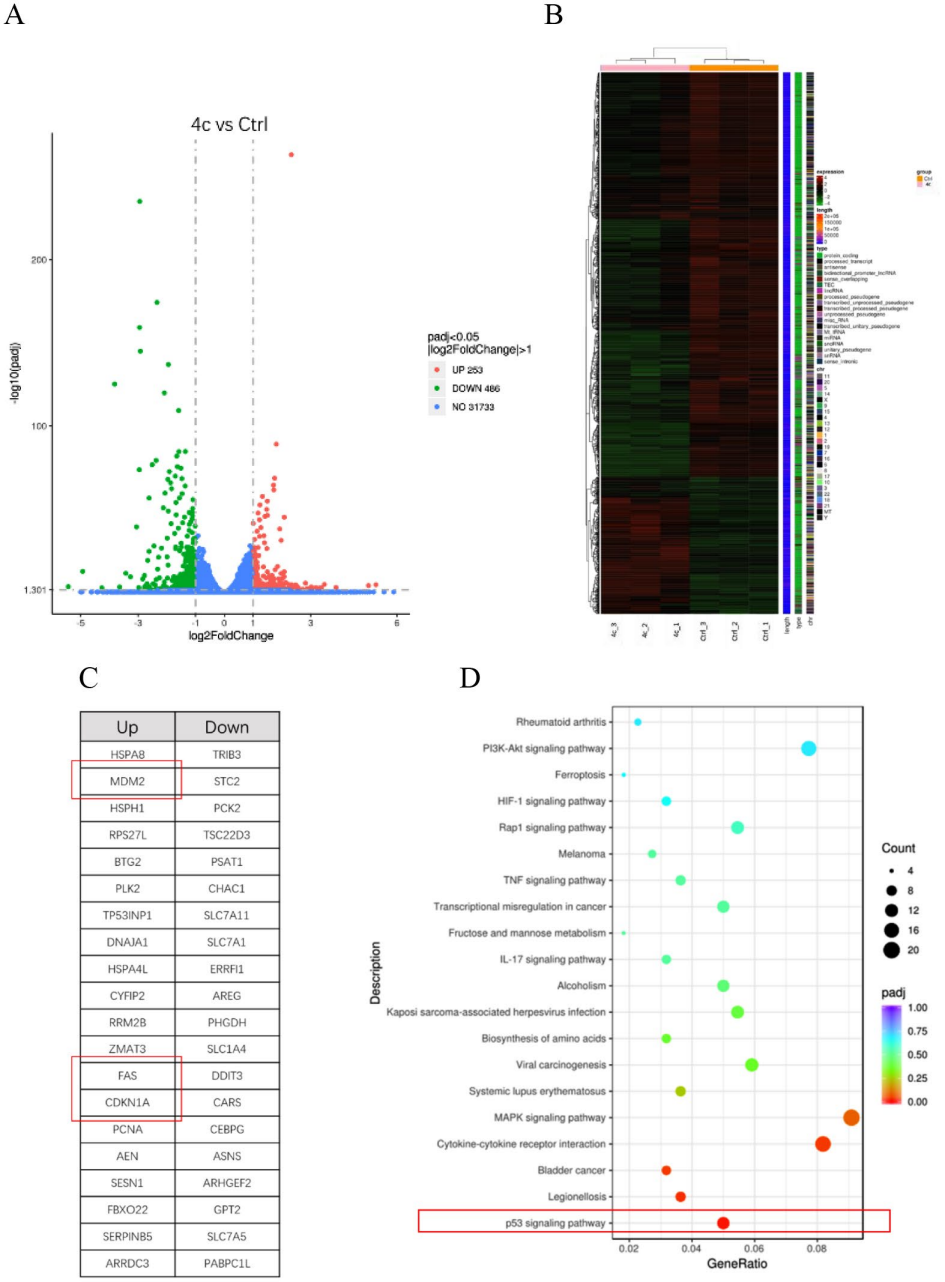
Compound 4c regulated cell cycle and FAS death receptor. **A**, volcano plot showing the number of differentially expressed genes between **4c** treatment group and the control group. **B**, heat map showing differentially expressed genes between **4c** treatment group and the control group. **C**, Top 20 up- and down-regulated genes after **4c** treatment, including MDM2, CDKN1A and FAS. **D**, KEGG analysis of the differentially expressed genes after **4c** treatment

To investigate the mechanism how **4c** exhibited its anticancer activity in CRC cell HCT116, RNA-seq (RNA sequencing) was conducted. Figure 5A revealed that 253 genes were significantly up-regulated while 486 genes were down-regulated after treatment of **4c**. Among the differentially expressed genes, the most up- and down-regulated genes were shown in Fig. 5B. The top 20 up- and down-regulated genes were listed in Fig. 5C. Focusing on cell proliferation and cell survival related genes, cell cycle-associated genes MDM2 and CDKN1A (also known as p21), as well as cell surface death receptor FAS were found to be significantly up-regulated. KEGG analysis suggested p53 signaling pathway was the most significantly regulated pathway (Fig. 5D).

### Compound 4c regulated cell cycle and FAS death receptor

**Fig. 6:**
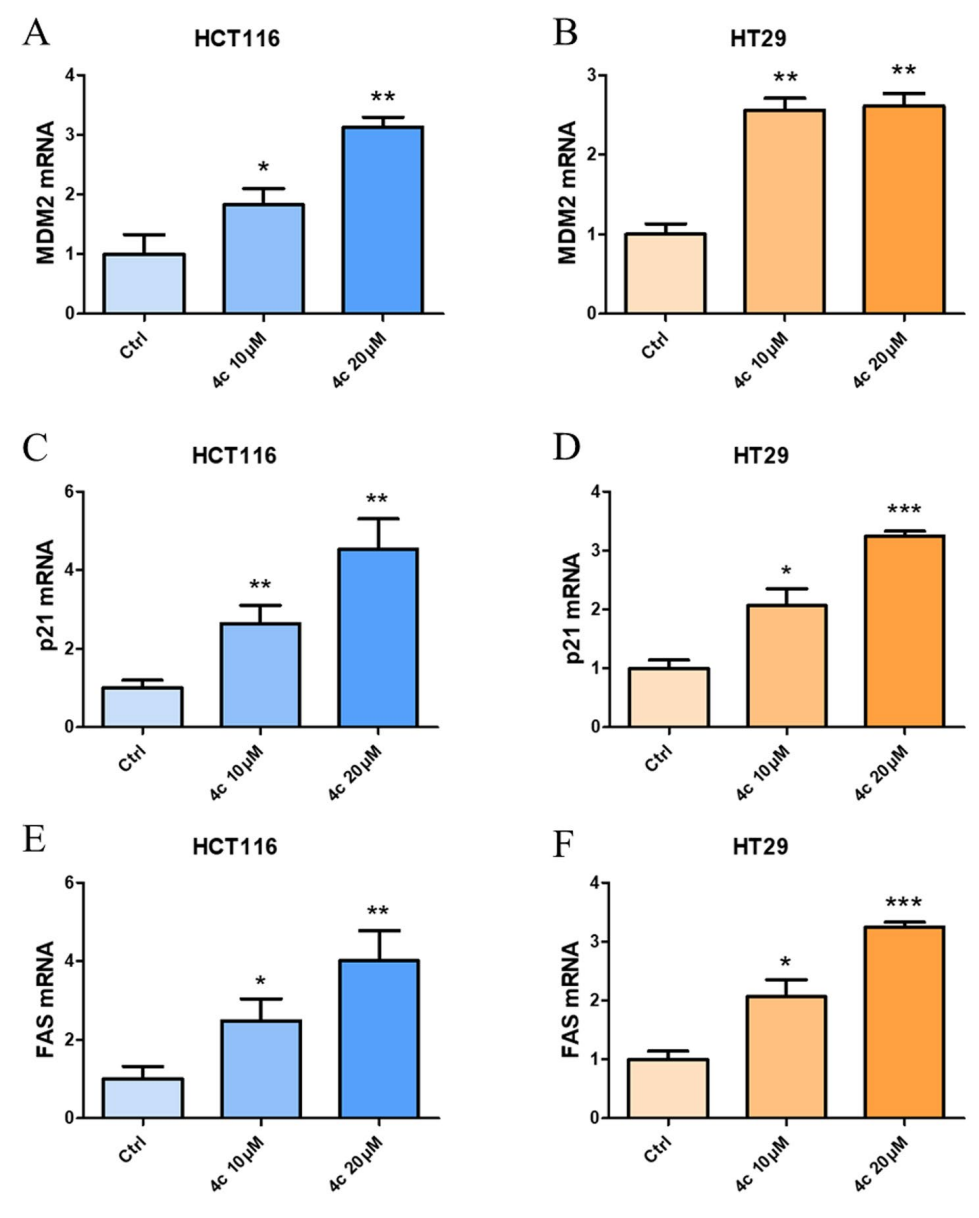
qPCR validation of MDM2, p21 and FAS expression in HCT116 and HT29 cells with treatment of compound 4c at various concentrations. Data are presented as mean ± SD. Statistically significant differences were indicated as *P < 0.05, **P < 0.01, ***P < 0.001

According to the result of RNA-seq analysis, validation experiment was conducted. qPCR indicated that the expression of cell cycle-associated genes MDM2 and p21, as well as cell surface death receptor FAS was significantly up-regulated both in HCT116 and HT29 cell lines with a dose-dependent manner (Fig. 6A–F). These results suggested **4c** probably exerted its anticancer activity by regulating cell cycle and FAS death receptor.

**Fig. 7:**
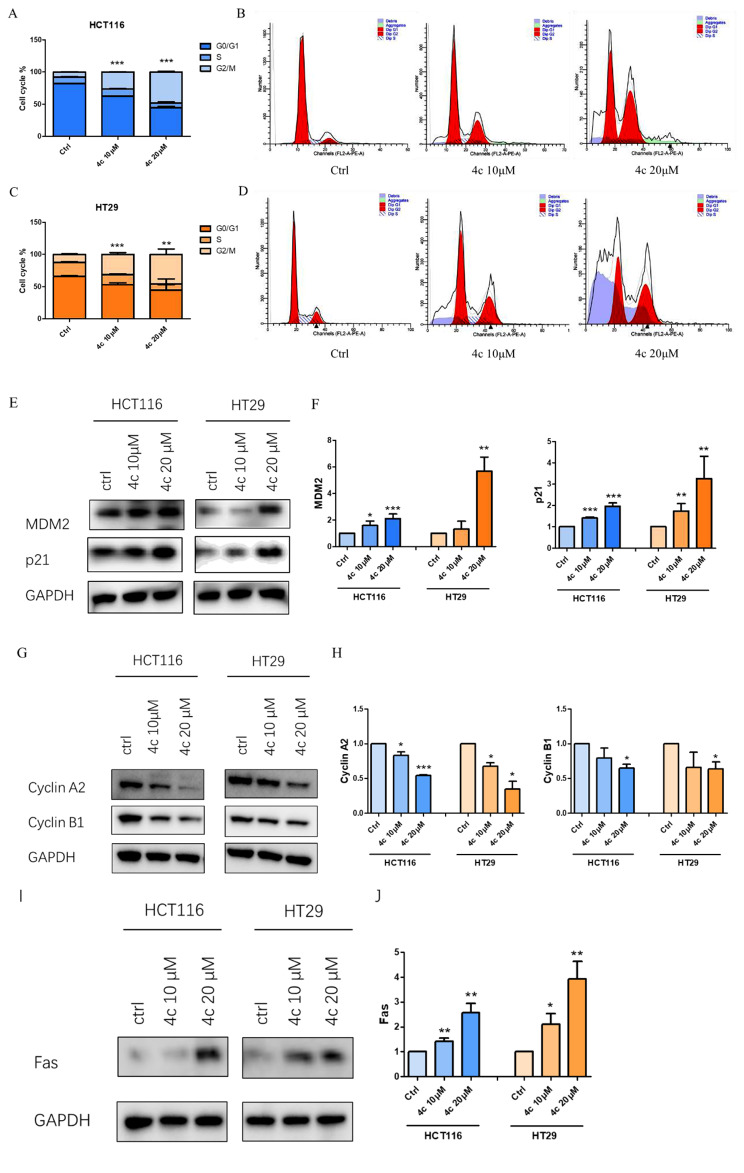
Compound 4c regulated cell cycle and FAS death receptor. **A**–**D**, cell cycle analysis of CRC celsl after **4c** treatment using flow cytometry. **E, F, 4c** promoted cell cycle regulator MDM2 and p21. **G, H, 4c** suppressed cell cycle checkpoints cyclin A2 and cyclin B. **I, J, 4c** up-regulated the expression of FAS death receptor. Data were presented as mean ± SD. Statistically significant differences were indicated as *P < 0.05, **P < 0.01, ***P < 0.001

To further confirm compound **4c** could regulate cell cycle and FAS death receptor, cell cycle analysis and western blotting was performed. Results showed **4c** could induce cell cycle G2/M arrest with a concentration-dependent manner (Fig. 7A–D). Meanwhile, western blotting results indicated that compound **4c** could also mediate cell cycle regulators MDM2 and p21 (Fig. 7E, F), as well as cell cycle G2/M checkpoints cyclin A2 and cyclin B1 (Fig. 7G, H). Meanwhile, **4c** could also up-regulate the expression of FAS cell surface death receptor, a member of the TNF-receptor family (Fig. 7I, J).

**Fig. 8:**
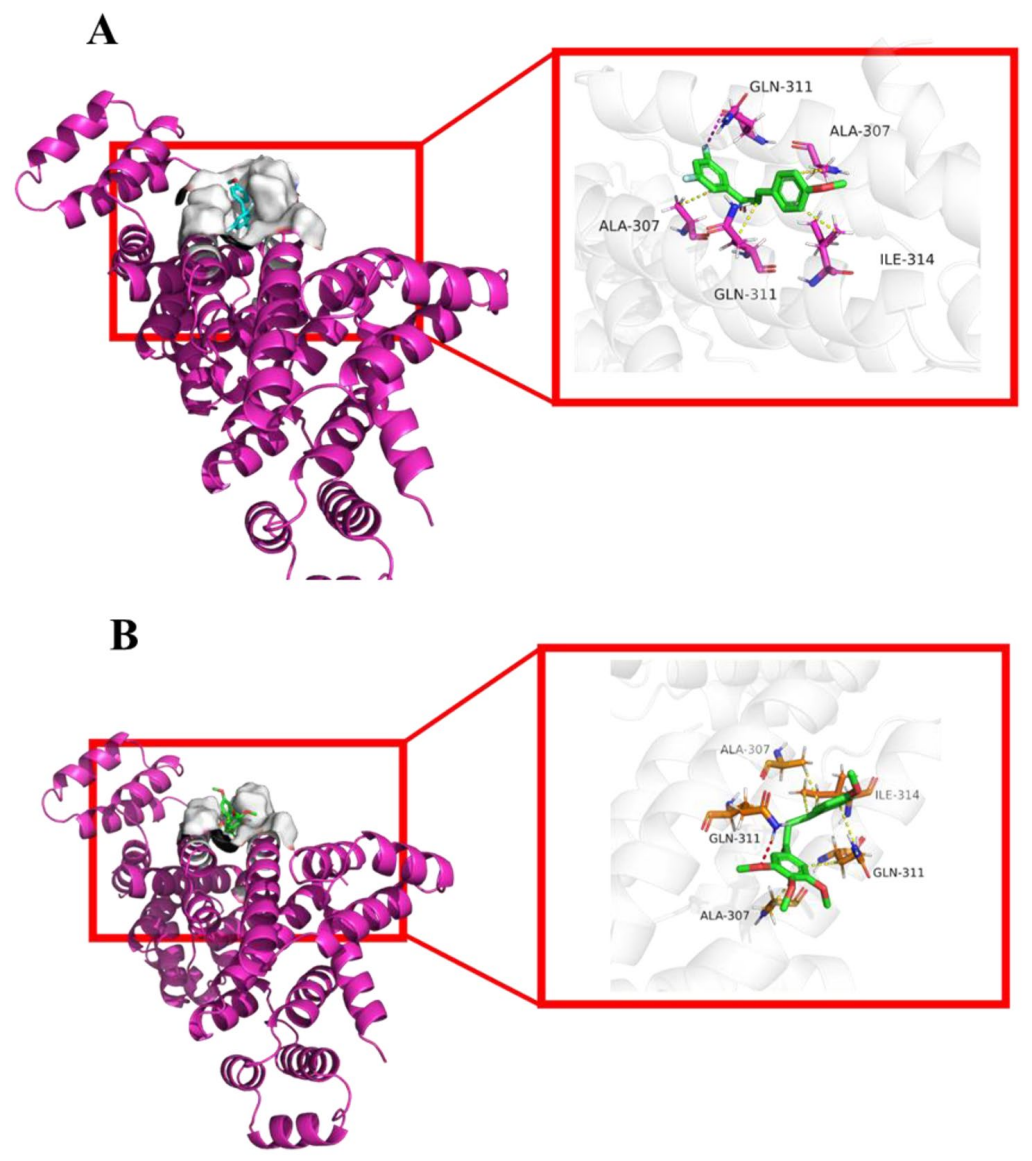
Binding mode of the Fas/FADD death domain complex (PDB code: 3EZQ) in complex with compounds **3d** (**A**) and **4c** (**B**)

AutoDock Vina was employed to provide more insights into molecular interactions between compound **3d** and the Fas/FADD (Fas-associated death domain protein) death domain complex (ID: 3EZQ), a tetrameric arrangement of four FADD death domains bound to four Fas death domains. The Fas-FADD death inducing signalling complex (DISC) assembled by Fas receptor, FADD and caspase 8 represents a receptor platform responsible for initiation of the induction of programmed cell death. The results shown in Fig. 8A revealed that the highest docking score positioned compound **3d** into the ligand-binding site of the Fas/FADD death domain complex. Hydrophobic interactions were predicted to occur between ALA-307 (A), ILE-314 (C), GLN-311 (C), ALA-307 (C) and compound **3d**. Furthermore, the docking score was −7.0, suggesting that **3d** had better binding energy with the Fas/FADD death domain complex.

PLIP website also figured out that compound **4c** formed hydrophobic interactions with residues ALA307 (A), ALA307 (C), GLN311 (A), and ILE314 (A) of the Fas/FADD death domain complex, as well as hydrogen bonds with GLN311 (C). Additionally, the distance of the abovementioned interactions ranged from 3.18 Å to 3.89 Å, suggesting strong physicochemical forces. Meanwhile, the binding affinity values of compound **4c** were −6.2 kcal/mol, indicating that **4c** nicely bound to the Fas/FADD death domain complex.

### Knockdown of FAS promoted the proliferation of CRC cells

**Fig. 9:**
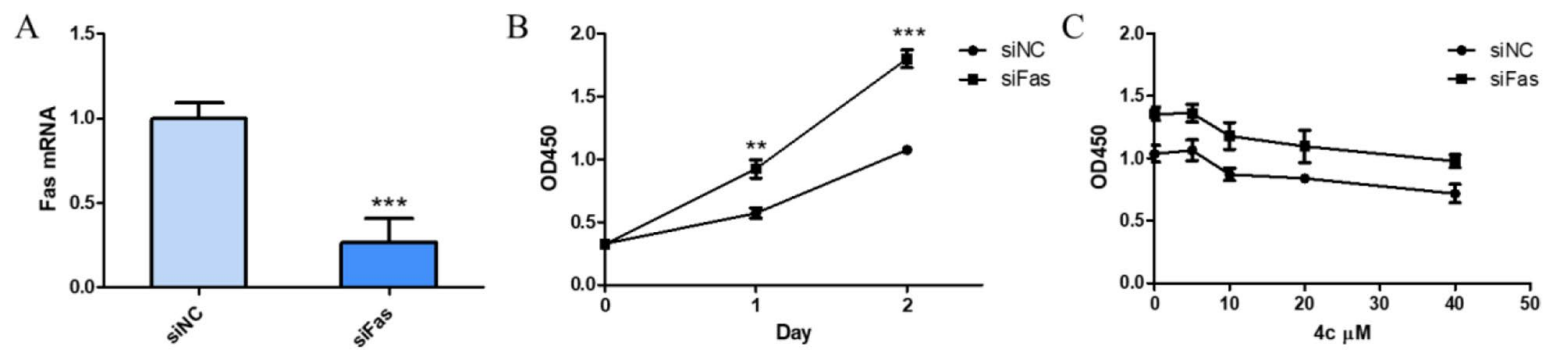
Knockdown of FAS promoted the proliferation of CRC cells. **A**, qPCR assay of HCT116 cell knocked down with FAS using siRNA. **B**, CCK-8 assay of HCT116 cell knocked down with FAS. **C**, CCK-8 assay of HCT116 cell knocked down with FAS followed by **4c** treatment for 24 h. Data were presented as mean ± SD. Statistically significant differences were indicated as *P < 0.05, **P < 0.01, ***P < 0.001

Since FAS was also known as apoptosis-associated gene, we then investigated whether **4c** could induce cytotoxicity by up-regulating FAS gene. Results showed that knockdown of FAS using small interfering RNA could significantly promote the proliferation of CRC cells (Fig. 9A, B). Moreover, HCT116 cells knocked down with FAS gene could attenuate the cytotoxicity induced by **4c** (Fig. 9C).

## Discussion

Loureirins are important bioactive components extracted from traditional Chinese medicine *Resina Draconis*. The anticancer activity of Loureirin analogues was investigated in this study. Preliminary screening results suggested Loureirin analogues possessed anti-CRC activity. Interestingly, SAR study suggested the α, β-unsaturated ketone of the chalcones **3** was crucial for their anticancer activity. A collection of reports also supported this hypothesis as chalcones possessed a ketoethylenic moiety which could serve as Michael acceptor in Michael addition reactions with the active site residues[32], leading to manifestation pharmacological potential of chalcones including antiproliferation, antibacterial, antivirus, antifibrotic, and anti-leishmania, etc. [33–37].

Among the Loureirin analogues screened in this study, chalcone **3d** and DHC **4c** exhibited specific anti-CRC activity meanwhile showed no cytotoxicity against human fetal lung fibroblast1 HFL1. Moreover, **4c** not only inhibited the proliferation but also the migration and invasion of CRC cells, suggesting **4c** exhibited better anti-malignant properties than **3d**. RNA-seq analysis and validation experiments indicated **4c** could regulate cell cycle regulator MDM2 and p21. Subsequent flow cytometry analysis confirmed **4c** could block cell cycle at G2/M phase. Cell cycle G2/M checkpoint cyclin A2 and cyclin B1 were also found to be significantly down-regulated by **4c** [12, 38].

Additionally, FAS was also found to be significantly up-regulated after **4c** treatment. FAS, a member of the TNFR family, is widely known to be involved in apoptosis pathway. It triggers apoptosis by binding with its ligand followed by the recruitment and assembly of the FAS DISC and subsequent activation of the caspase cascade [15, 16]. Other than apoptosis, FAS was also reported to regulate cell cycle G2/M arrest in CRC [39], which raised our hypothesis that **4c** probably induced cell cycle G2/M arrest by mediating FAS. While unfortunately, inconsistent with our hypothesis, knockdown of FAS gene did not regulate MDM2 and p21 significantly, and no reversing effect was observed even after **4c** treatment, from which we concluded that cell cycle G2/M arrest induced by **4c** was not mediated by FAS. As a result, **4c** exerted its anti-CRC activity by regulating cell cycle and possibly FAS-mediated apoptosis. Meanwhile, virtual docking also revealed that compound **4c** could nicely fitted into the receptor-binding site of the Fas/FADD death domain complex (ID: 3EZQ).

## Conclusion

To conclude, our research for the first time elucidated dihydrochalcone **4c**, inspired by Loureirin, important bioactive components extracted from traditional Chinese medicine *Resina Draconis*, exhibited selective anti-CRC activity via regulating cell cycle and FAS, which has the potential to be further developed into anticancer agents for the treatment of CRC.

## Material and methods

### Reagents, solvents and chemicals

Starting materials and reagents of commercial grade could be directly employed without further purification unless otherwise stated. All of the Loureirin analogues including chalcones **3** and DHCs **4** were synthesized according to our previous paper published [40]. All tested compounds with at least 95% purity were determined by an Agilent 1220 Infinity II. The NMR spectra, high resolution MS spectra, melting points, FT-IR spectrum and HPLC spectrum of the Loureirin analogues could be given with reference to the aforementioned paper whose DOI was 10.1039/D4MD00011K.

### Cell culture and transfection

Human cancer cell line HCT116, HT29 MCF7 was kindly provided by Dr. Ouyang from Shenzhen Polytechnic University and human embryonic lung fibroblast HFL1 was obtained from Shenzhen large-scaled cell culture and cell bank in Shenzhen Polytechnic University. Cells were cultured with RPMI 1640 medium (8121465, Gibco, USA), supplemented with 10 %v/v fetal bovine serum (FBS500-S, AusGeneX) and 1% penicillin/streptomycin (25200–056, Gibco, USA) at 37 °C in a saturated humidified atmosphere supplied with 5% CO_2_. Knockdown of Fas was performed by transient transfection with siRNA (RIBOBIO, China) and lipofectamine 2000 (2067563, Invitrogen, USA) according to the manufacturer’s instructions. The targeted sequence of Fas siRNA was GCAGATGTAAACCAAACTT. Negative control siRNA was obtained from RIBOBIO with catalogue No. siN0000001-1-5.

### CCK-8 assay and colony formation assay

According to our previous protocol published before, a number of 5000 cells/well was seeded in 96-well plate [41, 42]. Compounds were added after cell attachment with an incubation for 48 h. CCK-8 reagent (C6005, NCM Biotech, China) was used to determine cell proliferation according to the manufacturer’s instructions. OD450 was determined after CCK-8 incubation.

Colony formation was performed by seeding 2000 cells/well in 6-well plate and allowed to grow for 7 days followed by fixing. Fixing was conducted using methanol for 20 min at room temperature and staining was conducted using crystal violet for another 10 min.

### Cell migration and invasion assay

For cell migration assay, a number of 2 × 10^4^ cells/well of HCT116 cell was seeded into the upper chamber of the transwell insert (725301, NEST) in RPMI 1640 medium without FBS. The lower chamber was filled with RPMI 1640 medium with 10% FBS. Compound was added in culture medium in both the upper and lower chambers when cells were seeded. Cells were allowed to migrate for 24 h. Cells in the upper chamber of the insert were removed with cotton swabs and the migrated cells at the bottom of the membrane were fixed with methanol and stained with crystal violet. For cell invasion assay, the membrane of the transwell insert was coated with Matrigel (354234, Corning,) on top according to the manufacturer’s instructions. A number of 4 × 10^4^ cells/well of HCT116 cells was used. Cells were allowed to invade for 48 h.

### Western blot

Cells with different treatment were lysed using RIPA lysis bufer (WB3100, NCM Biotech, China) supplemented with 1 × protease inhibitor (70090050, Biosharp, China) and 1 × phosphatase inhibitor (70080020, Biosharp, China) at 4 °C. Western blot was performed according to the common protocol. The primary antibodies used were anti-MDM2 antibody (ab3110, abcam), anti-p21 antibody (2947T, CST), anti-Fas antibody (4233T, CST), anti-GAPDH antibody (5174S, CST), anti-cyclin A2 antibody (4656S, CST), anti-cyclin B1 antibody (12231S, CST). The secondary antibodies used were Anti-mouse IgG, HRP-linked Antibody (7076S, CST) and Anti-rabbit IgG, HRP-linked Antibody (7074S, CST). The dilution for primary antibody was 1:1000 and the dilution for secondary antibody was 1:3000.

#### Flow cytometry

A number of 6 × 10^5^ cells/well were seeded in 6-well plate for 24 h before drug treatment. HCT116 cell was treated with 4c for 48 h and HT29 cell was treated with 4c for 24 h before cell collection followed by cell fixation with 70% ethanol. Cells were stained with cell cycle analysis kit from BD (550825). Flow cytometry analysis was performed using Beckman DxFLEX flow cytometer. Data analysis was conduceted using modfit.

### RNA extraction and quantitative PCR (qPCR)

Extraction was conducted using RNA extraction kit (DP419, DP501, Tiangen, China). Reverse transcription was conducted using reverse transcription kit (RR037A, Takara). qPCR was performed using StepOnePlus (ABI) and SYBR green qPCR kit (A6002, Promega).

Primers used in the qPCR assays were

- GAPDH forward: 5′-CGAGATCCCTCCAAAATCAA-3′,
- GAPDH reverse: 5′-GGTGCTAAGCAGTTGGTGGT-3′,
- FAS forward: 5′-GACTCAGAACTTGGAAGGCC-3′,
- FAS reverse: 5′-TCATGACTCCAGCAATAGTGG-3′,
- p21 forward: 5′-TAGCAGCGGAACAAGGAG-3′,
- p21 reverse: 5′-AAACGGGAACCAGGACAC-3′,
- MDM2 forward: 5′-TTATTAAAGTCTGTTGGTGCA-3′,
- MDM2 reverse: 5′- TGAAGGTTTCTCTTCCTGAAG-3′.

### RNA-seq

HCT116 cell was seeded in 3-cm dish followed by 20 μM of **3c** treatment for 24 h before RNA extraction. Samples were prepared in triplicates. Total RNA was extracted with Trizol reagent (15596026, Invitrogen) according to the manufacture’s instructions. Preparation of RNA library and transcriptome sequencing was conducted by Novogene Co., LTD (Beijing, China). Genes with adjusted *p*-value < 0.05 and|log2(FoldChange)| > 1 were set as the threshold for significantly differential expression.

### Computational docking studies

AutoDock vina 1.2.0 was employed for virtual docking [43, 44]. The 3D structures of compounds **3d** and **4c** were provided and conducted energy minimization by ChemDraw 22.0 prior to importing into the AutoDock Vina. The crystal structure of the Fas/FADD Death Domain Complex (ID: 3EZQ) downloaded from the Protein Data Bank (https://www.rcsb.org/) was used for docking measurments after removal of water molecules, metal ions, and small ligands bound to the protein, as well as the duplicate domains. According to the papers reported before, the ligand-binding pocket of the Fas/FADD Death Domain Complex was thought to be around the crucial residue Ile313, necessary for the Fas-opening which is a key step responsible for the formation of Fas–Fas dimer and the receptor–adaptor interactions [45, 46]. The binding results after analysis of PLIP website were visualized by PyMOL version 2.5.2 software.

### Statistical analysis

All the quantitative results were presented as the mean ± standard deviation (SD). Student’s *t*-test was used to compare the Statistical significance between two groups using Prism. Differences were considered as significant when *P < 0.05, **P < 0.01, ***P < 0.001.

### Electronic supplementary material

Below is the link to the electronic supplementary material.

Supplementary Material 1

## Data Availability

The datasets used and/or analyzed during the current study are available from the corresponding author on reasonable request.

## Abbreviations

SAR: Structure-activity relationships
DHC: Dihydrochalcone
RNA-seq: RNA sequencing
TNFR: TNF receptor
DISC: Death-inducing signaling complex

## References

[1] Sung H, Ferlay J, Siegel RL, Laversanne M, Soerjomataram I, Jemal A, Bray F (2021). Global cancer statistics 2020: GLOBOCAN estimates of incidence and mortality worldwide for 36 cancers in 185 countries. CA Cancer J Clinicians.

[2] Engstrand J, Nilsson H, Strömberg C, Jonas E, Freedman J (2018). Colorectal cancer liver metastases– a population-based study on incidence, management and survival. BMC Cancer.

[3] Piawah S, Venook AP (2019). Targeted therapy for colorectal cancer metastases: a review of current methods of molecularly targeted therapy and the use of tumor biomarkers in the treatment of metastatic colorectal cancer. Cancer.

[4] McQuade MR, Stojanovska V, Bornstein CJ, Nurgali K (2017). Colorectal cancer chemotherapy: the evolution of treatment and new approaches. Curr Med Chem.

[5] Bertino JR (1997). Chemotherapy of colorectal cancer: history and new themes. Semin Oncol.

[6] Chibaudel B, Tournigand C, Bonnetain F, Richa H, Benetkiewicz M, André T, de Gramont A (2015). Therapeutic strategy in unresectable metastatic colorectal cancer: an updated review. Ther Adv Med Oncol.

[7] Xie Y-H, Chen Y-X, Fang J-Y (2020). Comprehensive review of targeted therapy for colorectal cancer. Signal Transduct Target Ther.

[8] Marcus L, Lemery SJ, Keegan P, Pazdur R (2019). FDA approval summary: pembrolizumab for the treatment of microsatellite instability-high solid tumors. Clin Cancer Res.

[9] Mármol I, Sánchez-de-diego C, Pradilla Dieste A, Cerrada E, Rodriguez Yoldi MJ (2017). Colorectal carcinoma: a general overview and future perspectives in colorectal cancer. Int J Mol Sci.

[10] Cai S, Moutal A, Yu J, Chew LA, Isensee J, Chawla R, Gomez K, Luo S, Zhou Y, Chefdeville A, Madura C, Perez-Miller S, Bellampalli SS, Dorame A, Scott DD, François-Moutal L, Shan Z, Woodward T, Gokhale V, Hohmann AG, Vanderah TW, Patek M, Khanna M, Hucho T, Khanna R (2021). Selective targeting of NaV1.7 via inhibition of the CRMP2-Ubc9 interaction reduces pain in rodents. Sci Transl Med.

[11] Wang Z, Wang Z (2022). Cell cycle progression and synchronization: an overview. Cell-cycle Synchronization: methods and Protocols.

[12] Gillis LD, Leidal AM, Hill R, Lee PWK (2009). p21Cip1/WAF1 mediates cyclin B1 degradation in response to DNA damage. Cell Cycle.

[13] Visanji JM, Thompson DG, Padfield PJ (2006). Induction of G2/M phase cell cycle arrest by carnosol and carnosic acid is associated with alteration of cyclin A and cyclin B1 levels. Cancer Lett.

[14] Kamarudin MNA, Sarker MMR, Zhou J-R, Parhar I (2019). Metformin in colorectal cancer: molecular mechanism, preclinical and clinical aspects. J Exp Clin Cancer Res.

[15] Green DR (2022). The death receptor pathway of apoptosis. Cold Spring Harb Perspect Biol.

[16] Lavrik IN (2014). Systems biology of death receptor networks: live and let die. Cell Death Dis.

[17] Mollinedo F, Gajate C (2006). Fas/CD95 death receptor and lipid rafts: new targets for apoptosis-directed cancer therapy. Drug Resist Updates.

[18] Zhuang C, Zhang W, Sheng C, Zhang W, Xing C, Miao Z (2017). Chalcone: a privileged structure in medicinal chemistry. Chem Rev.

[19] Ouyang Y, Li J, Chen X, Fu X, Sun S, Wu Q (2021). Chalcone derivatives: role in anticancer therapy. Biomolecules.

[20] Henry EJ, Bird SJ, Gowland P, Collins M, Cassella JP (2020). Ferrocenyl chalcone derivatives as possible antimicrobial agents. Journal Antibiot..

[21] Polo E, Ibarra-Arellano N, Prent-Peñaloza L, Morales-Bayuelo A, Henao J, Galdámez A, Gutiérrez M (2019). Ultrasound-assisted synthesis of novel chalcone, heterochalcone and bis-chalcone derivatives and the evaluation of their antioxidant properties and as acetylcholinesterase inhibitors. Bioorg Chem.

[22] Tang YL, Zheng X, Qi Y, Pu XJ, Liu B, Zhang X, Li XS, Xiao WL, Wan CP, Mao ZW (2020). Synthesis and anti-inflammatory evaluation of new chalcone derivatives bearing bispiperazine linker as IL-1β inhibitors. Bioorg Chem.

[23] Pinto P, Machado CM, Moreira J, Almeida JDP, Silva PMA, Henriques AC, Soares JX, Salvador JAR, Afonso C, Pinto M, Bousbaa H, Cidade H (2019). Chalcone derivatives targeting mitosis: synthesis, evaluation of antitumor activity and lipophilicity. Eur J Med Chem.

[24] Rivière C, Atta ur R (2016). Chapter 7 - Dihydrochalcones: occurrence in the plant kingdom, chemistry and biological activities. Studies in Natural Products Chemistry.

[25] Dierckx T, Vanherle S, Haidar M, Grajchen E, Mingneau F, Gervois P, Wolfs E, Bylemans D, Voet A, Nguyen T, Hamad I, Kleinewietfeld M, Bogie JFJ, Hendriks JJA (2022). Phloretin enhances remyelination by stimulating oligodendrocyte precursor cell differentiation. Proc Natl Acad Sci USA.

[26] Kamdi SP, Badwaik HR, Raval A, Ajazuddin, Nakhate KT. Ameliorative potential of phloridzin in type 2 diabetes-induced memory deficits in rats. Eur J Pharmacol. 2021;913:174645. 10.1016/j.ejphar.2021.174645 34800467

[27] Su XQ, Song YL, Zhang J, Huo HX, Huang Z, Zheng J, Zhang Q, Zhao YF, Xiao W, Li J, Tu PF (2014). Dihydrochalcones and homoisoflavanes from the red resin of Dracaena cochinchinensis (Chinese dragon’s blood). Fitoterapia.

[28] Shi S, Zhao Q, Ke C, Long S, Zhang F, Zhang X, Li Y, Liu X, Hu H, Yin S (2021). Loureirin B exerts its immunosuppressive effects by inhibiting STIM1/Orai1 and K(V)1.3 channels. Front Pharmacol.

[29] Shi W, Hu J, Bao N, Li D, Chen L, Sun J (2017). Design, synthesis and cytotoxic activities of scopoletin-isoxazole and scopoletin-pyrazole hybrids. Bioorg Med Chem Lett.

[30] Ahmed HEA, El-Nassag MAA, Hassan AH, Okasha RM, Ihmaid S, Fouda AM, Afifi TH, Aljuhani A, El-Agrody AM (2018). Introducing novel potent anticancer agents of 1H-benzo[f]chromene scaffolds, targeting c-Src kinase enzyme with MDA-MB-231 cell line anti-invasion effect. J Enzyme Inhib Med Chem.

[31] Grozav A, Balacescu O, Balacescu L, Cheminel T, Berindan-Neagoe I, Therrien B (2015). Synthesis, anticancer activity, and genome profiling of thiazolo arene ruthenium complexes. J Med Chem.

[32] Jackson PA, Widen JC, Harki DA, Brummond KM (2017). Covalent modifiers: a chemical perspective on the reactivity of α,β-unsaturated carbonyls with thiols via hetero-Michael addition reactions. J Med Chem.

[33] Elkanzi NAA, Hrichi H, Alolayan RA, Derafa W, Zahou FM, Bakr RB (2022). Synthesis of chalcones derivatives and their biological activities: a review. ACS Omega.

[34] Dan W, Dai J (2020). Recent developments of chalcones as potential antibacterial agents in medicinal chemistry. Eur J Med Chem.

[35] Poolsri W, Noitem R, Jutabha P, Raveesunthornkiat M, Danova A, Chavasiri W, Muanprasat C (2023). Discovery of a chalcone derivative as an anti-fibrotic agent targeting transforming growth factor-β1 signaling: potential therapy of renal fibrosis. Biomed Pharmacother.

[36] de Santiago-silva KM, da Silva Gomes GF, Perez CC, da Silva Lima CH, de Lima Ferreira Bispo M (2023). Molecular targets for chalcones in antileishmanial drug discovery. Mini Rev Med Chem.

[37] Elkhalifa D, Al-Hashimi I, Al Moustafa AE, Khalil A (2021). A comprehensive review on the antiviral activities of chalcones. J Drug Targeting.

[38] Stark GR, Taylor WR, Schönthal AH (2004). Analyzing the G2/M checkpoint. Checkpoint Controls and Cancer: volume 1: reviews and Model Systems.

[39] Park Y-L, Ha S-Y, Park S-Y, Choi J-H, Jung M-W, Myung D-S, Kim H-S, Joo Y-E (2019). Reversine induces cell cycle arrest and apoptosis via upregulation of the Fas and DR5 signaling pathways in human colorectal cancer cells. Int J Oncol.

[40] Zhang D, Wang W, Ou H, Ning J, Zhou Y, Ke J, Hou A, Chen L, Li P, Ma Y, Jin W. Identification of chalcone analogues as anti-inflammatory agents through regulation of NF-κB and JNK activation. RSC Med Chem 2024. 10.1039/d4md00011k PMC11187561 38911149

[41] Yang W, Wang W, Cai S, Li P, Zhang D, Ning J, Ke J, Hou A, Chen L, Ma Y, Jin W (2023). Synthesis and in vivo antiarrhythmic activity evaluation of novel scutellarein analogues as voltage-gated Nav1.5 and Cav1.2 channels blockers. Molecules (Basel, Switzerland).

[42] Jin W, Xu C, Dong N, Chen K, Zhang D, Ning J, Li Y, Zhang G, Ke J, Hou A, Chen L, Chen S, Chan K-F (2023). Identification of isothiazolones analogues as potent bactericidal agents against antibiotic resistant CRE and MRSA strains. BMC Chem.

[43] Eberhardt J, Santos-Martins D, Tillack AF, Forli S (2021). AutoDock Vina 1.2.0: new docking methods, expanded force field, and python bindings. J Chem Inf Model.

[44] Trott O, Olson AJ (2010). AutoDock Vina: improving the speed and accuracy of docking with a new scoring function, efficient optimization, and multithreading. J Comput Chem.

[45] Scott FL, Stec B, Pop C, Dobaczewska MK, Lee JJ, Monosov E, Robinson H, Salvesen GS, Schwarzenbacher R, Riedl SJ (2009). The Fas-FADD death domain complex structure unravels signalling by receptor clustering. Nature.

[46] Roy U (2016). Structural characterizations of the Fas receptor and the Fas-associated protein with death domain interactions. Protein J.

