# Supplementary material for: Discovery of Loureirin analogues with colorectal cancer suppressive activity via regulating cell cycle and Fas death receptor
Source: BMC Pharmacol Toxicol. 2024 Jun 28;25:36. doi: 10.1186/s40360-024-00758-2 (PMC11212204; doi:10.1186/s40360-024-00758-2)

Figure 7C

MDM2

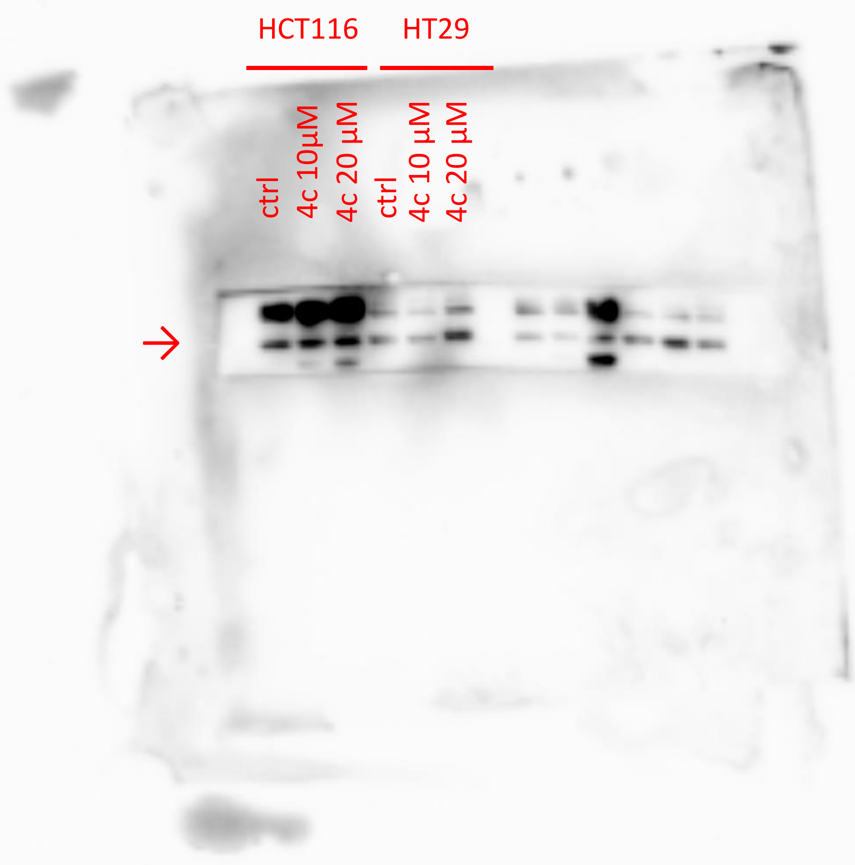

GAPDH

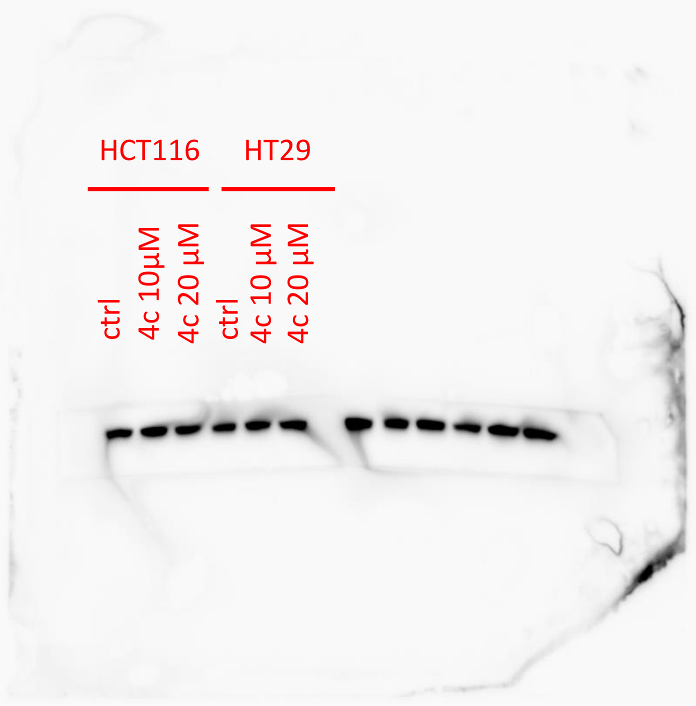

Figure 7C

p21
Exposure weak for HCT116

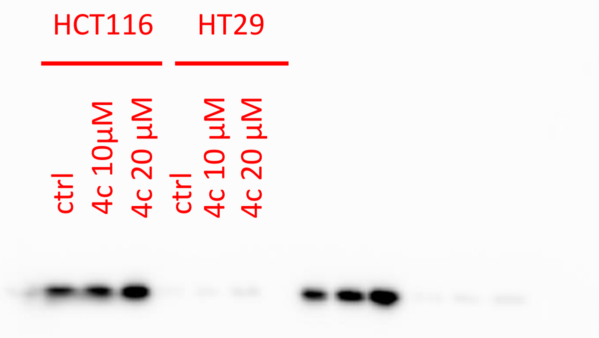

p21
Exposure strong for HT29

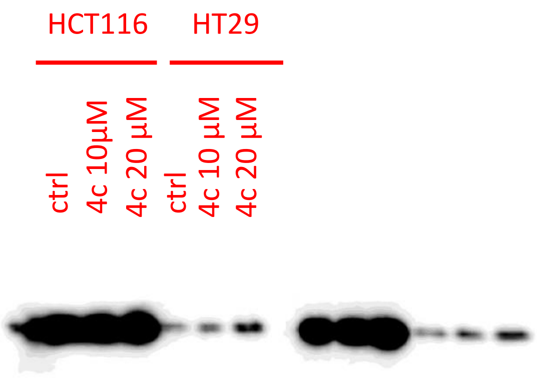

Figure 7D

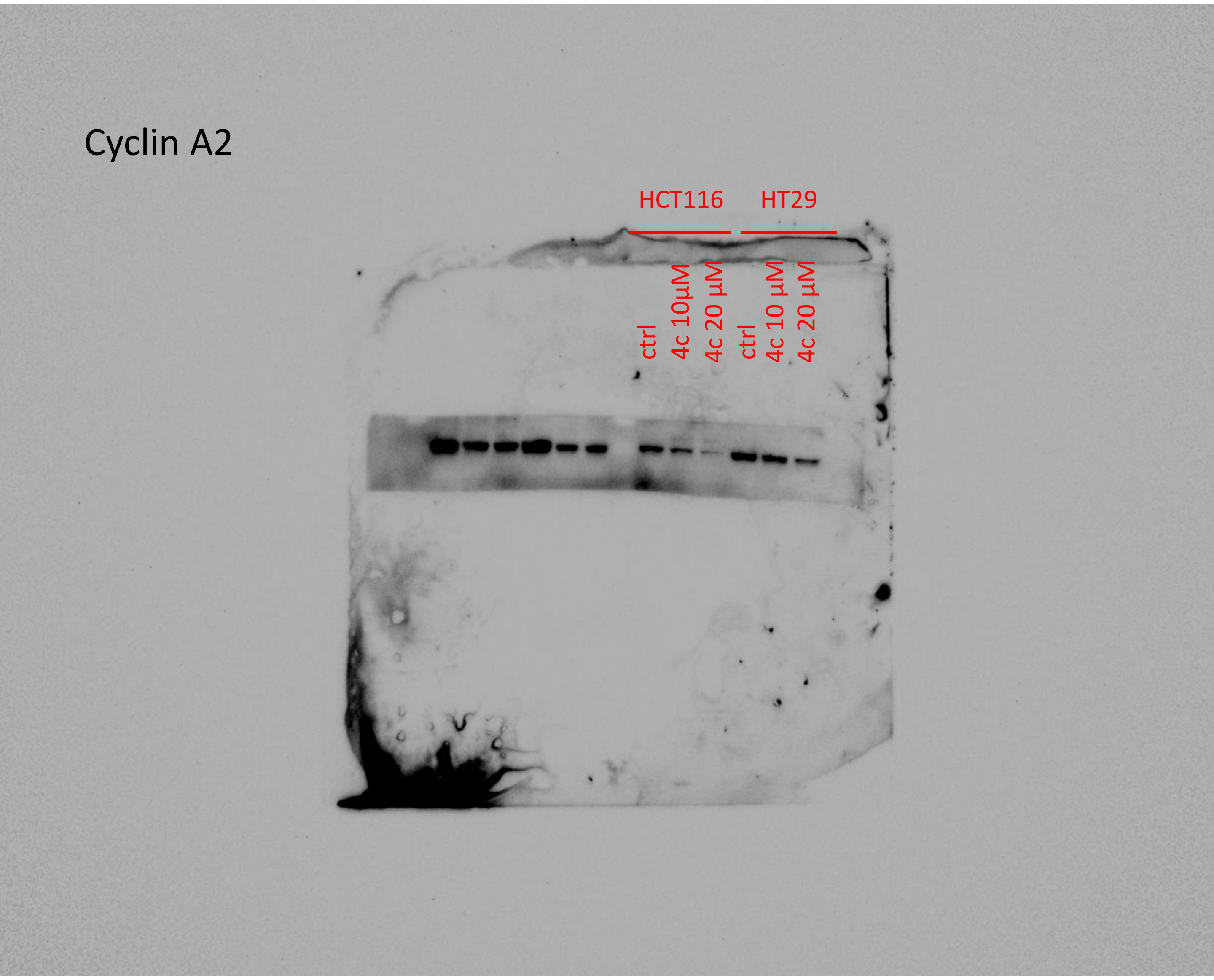
Cyclin A2
HCT116
HT29
ctrl
4c 10µM
4c 20 µM
ctrl
4c 10 µM
4c 20 µM

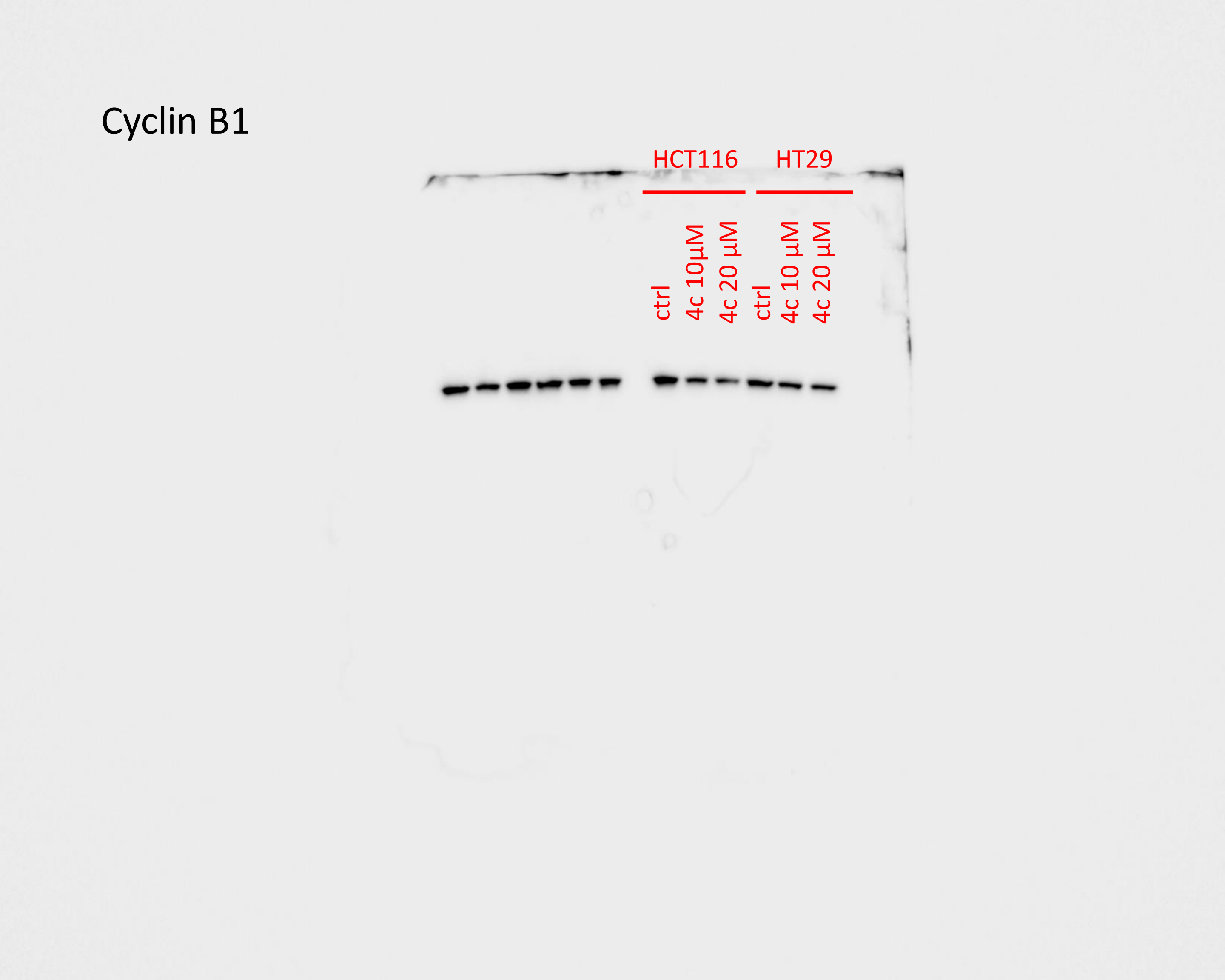
Cyclin B1
HCT116
HT29
ctrl
4c 10µM
4c 20 µM
ctrl
4c 10 µM
4c 20 µM

Figure 7D

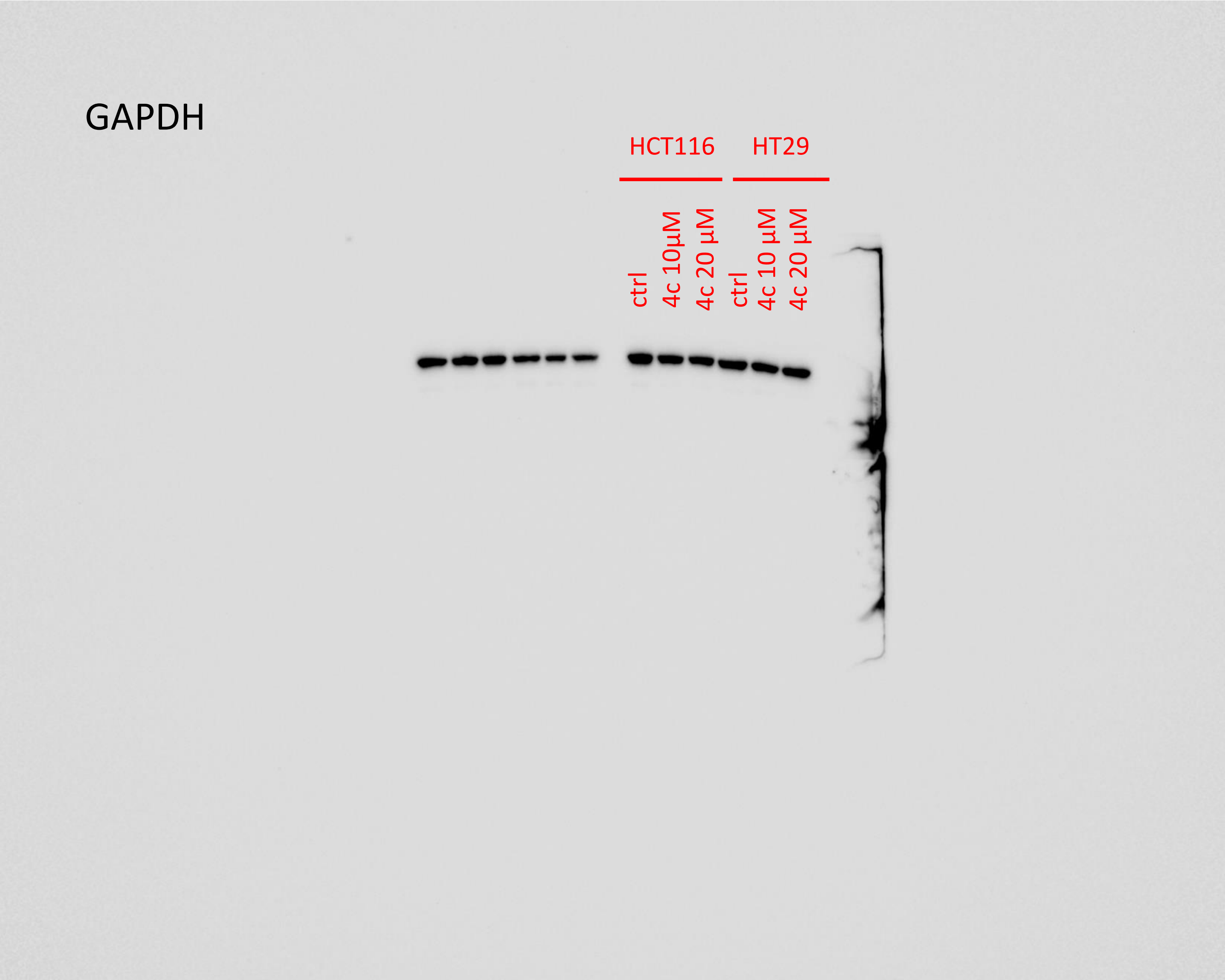

Figure 7E

Fas
Exposure weak for HCT116

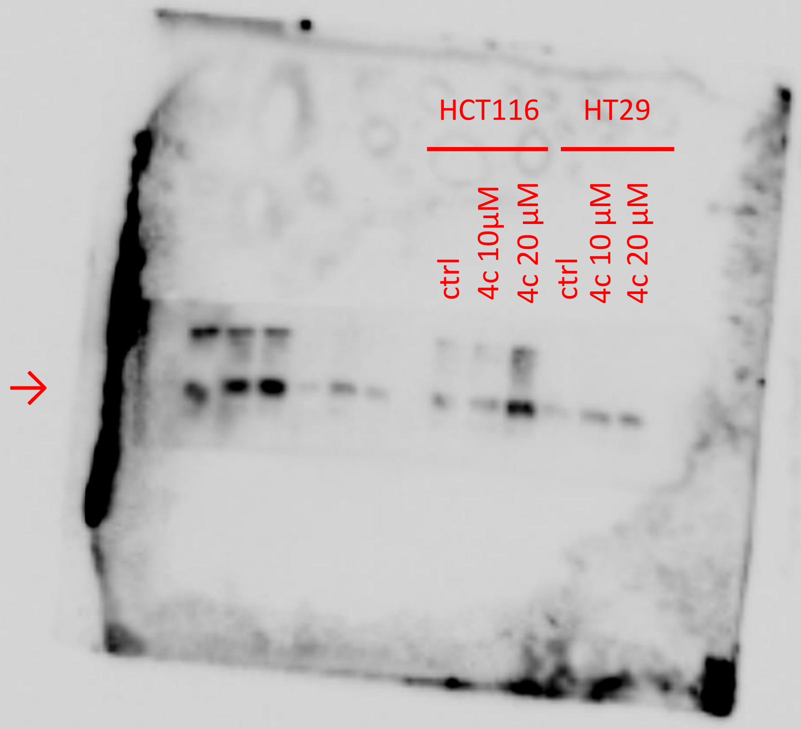

Fas
Exposure strong for HT29

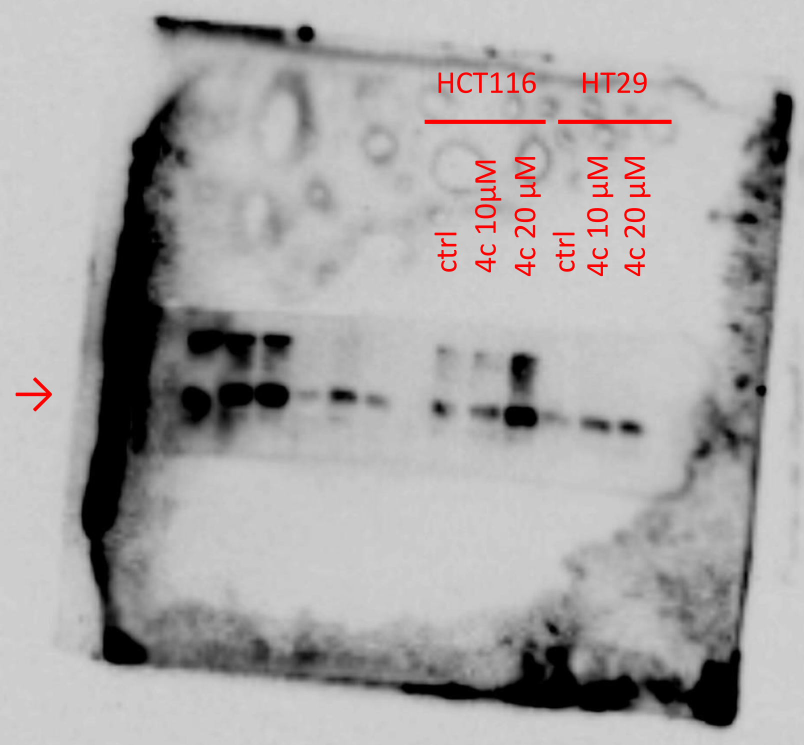

Figure 7E

GAPDH

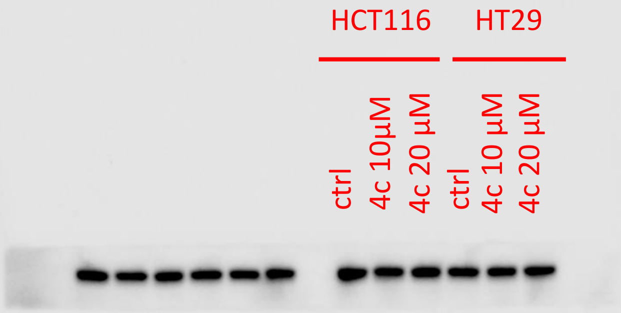

Supplement: Supplementary file 1 — Supplementary Material 1 [file 40360_2024_758_MOESM1_ESM.pdf]
